# Is Whip Use Important to Thoroughbred Racing Integrity? What Stewards’ Reports Reveal about Fairness to Punters, Jockeys and Horses

**DOI:** 10.3390/ani10111985

**Published:** 2020-10-29

**Authors:** Kirrilly Thompson, Phil McManus, Dene Stansall, Bethany J. Wilson, Paul D. McGreevy

**Affiliations:** 1 University of South Australia, Adelaide, SA 5034, Australia; 2 School of Geosciences, University of Sydney, Sydney, NSW 2006, Australia; phil.mcmanus@sydney.edu.au; 3 Dene Stansall, Animal Aid, The Old Chapel, Bradford Street, Tonbridge, Kent TN9 1AW, UK; dene@animalaid.co.uk; 4 School of Veterinary Science, University of Sydney, Sydney, NSW 2006, Australia; bethany.wilson@sydney.edu.au (B.J.W.); paul.mcgreevy@sydney.edu.au (P.D.M.)

**Keywords:** jockeys, safety, steering, risk, social license to operate, public perception, integrity

## Abstract

**Simple Summary:**

As a multibillion-dollar industry involving gambling and animals, fairness is essential to thoroughbred racing. This is referred to as racing integrity. Whilst there are comprehensive rules and regulations governing equipment and conduct, whip use is the most publicly visible enforcement of integrity in racing. As a tool for “encouragement”, whip use is believed to give everyone a fair chance of winning, including owners, trainers, jockeys, horses and punters. As a tool for “steering”, whip use is also believed to be essential for the safety of the horse and jockey. However, the impact of whip use on steering and safety has not been studied. In this article, we compare “whipping-free” races in Great Britain, where whips are held but not used with the more commonplace “whipping-permitted” races. Our analysis of stewards’ reports for 126 races involving 1178 starters over three years found no statistically significant differences between stewards having anything to report, movement on course, interference on course, incidents related to jockey behaviour or race finishing times. Our findings, that whip use is not related to racing integrity, support the normalisation of “whipping-free” races, which we expect to improve horse welfare and social acceptance.

**Abstract:**

The idea that whip use is critical to thoroughbred racing integrity is culturally entrenched but lacks empirical support. To test the longstanding beliefs that whip use aids steering, reduces interference, increases safety and improves finishing times, we conducted a mixed-method analysis of 126 race reports produced by official stewards of the British Horseracing Authority, representing 1178 jockeys and their horses. We compared reports from 67 “Hands and Heels” races, where whips are held but not used (whipping-free, WF), with 59 reports from case-matched races where whipping was permitted (whipping permitted, WP). Qualitative coding was used to identify and categorise units of analysis for statistical testing via logistic regression and linear mixed model regression. For both types of race, we explored stewards having anything to report at all, movement on course, interference on course, incidents related to jockey behaviour and finishing times. There were no statistically significant differences between WF and WP races for anything to report (OR: 3.06; CI: 0.74–14.73), movement on course (OR: 0.90; CI: 0.37–2.17), interference (OR: 0.90; CI: 0.37–2.17), jockey-related incidents (OR: 1.24; CI: 0.32–5.07), and race times (0.512 s, *t* = 1.459, *p* = 0.150). That is, we found no evidence that whip use improves steering, reduces interference, increases safety or improves finishing times. These findings suggest that the WF races do not compromise racing integrity. They also highlight the need for more effective ways to improve the steering of horses.

## 1. Introduction

Professional thoroughbred racing is a public sport with a global audience. As with any competitive sport, fairness is paramount, and rules and regulations have been developed to create a level playing field for all participants. In horseracing, the importance of fairness is heightened by sports betting/gambling, which in leading horseracing nations is a multi-billion-dollar industry [1]. Punters have an expectation that the rules are adhered to by all participants and are strictly enforced and that no corruption or unfair advantage has affected the odds of a win or place. That is, they expect a proverbial “good, clean fight”, as do all racehorse breeders, trainers, owners, spectators and jockeys. The involvement of animals in a sport that takes place for the purposes of human financial gain adds another layer of complexity to fairness in horseracing. This extends to issues of equine welfare, but also includes moral and ethical debate, which can jeopardize an industry’s social licence to operate (SLO) [2].

Racing integrity is the term that is frequently used to encompass ideas of fairness in relation to the aforementioned spectacle, sport, gambling and animal welfare issues related to thoroughbred horseracing. Integrity is central to horseracing’s SLO, hence the national organisations responsible for the regulation of horseracing have teams, protocols, rules, regulations and stewards dedicated to upholding integrity in racing. The reach of such measures is broad, encompassing compliance during training and at official racing venues, horse/jockey doping, betting practices, corruption and inside information. Integrity also includes equine welfare, although that is largely limited to basic equine health and functioning [3].

Racing integrity is the most demonstrable factor and is subject to public scrutiny during an actual race. Once a race is in progress, every horse is expected to be given a fair opportunity to win. Specifically, this means that there should be no interference between horses and that all horses in contention should be ridden out on their merits, regardless of the perceived likelihood of a placing. The whip has been considered important for on-course integrity for two main reasons. First, the whip is believed to support a jockey’s ability to steer, which is necessary to avoid interference between horses [4]. Given that interference can lead to serious or catastrophic injury, whip use is therefore also perceived to be essential for horse and jockey safety. Second, use of the whip is taken as evidence that jockeys are meeting their obligation to ride horses out on their full merits. This is important to counter accusations that a horse was not given full opportunity to win or place. In these instances, whip use if often referred to as “encouragement” or “persuasion” for the horse, as can be seen in the following statement from the British Horseracing Authority (BHA) website:

“The use of the whip in British racing is restricted to safety, correction and encouragement. By ‘encouragement’ we mean using the whip as an aid to activate and focus the horse, so the horse realises its potential by giving its best. Use of the whip to coerce is not permitted, and the rules are designed to reflect this” BHA [5].

Despite the BHA rules to restrict and limit whip use Rule F(45) [6], the line between encouragement and coercion can become blurred, especially given the pressures on jockeys to demonstrate that they have met their obligations to racing integrity by giving the horse all the necessary encouragement to win. The importance of riding a horse out on its merits is reflected in the imposition of at least two days of suspension for jockeys found guilty of “[f]ailing to ride out approaching the finish on a horse that would have been placed” to three years for “[i]ntentionally not riding a horse on its merits that would have won” Rule F(37) [6]. The practice, known more commonly as the jockey “dropping their hands”, is taken to mean that the jockey has failed in their duty to ride the horse out on its merits. This permits critique that the horse may have otherwise won or placed.

There are many ways to uphold racing integrity such as via swabbing, closed circuit TV monitoring and the full gamut of actions, processes, policies and practices. However, none are more visible or salient to the public than whip strikes. Given that integrity and safety are critical, it is hardly surprising that whip use has been normalized in horseracing. Only Norway prohibits the carrying of whips in all flat races involving horses three years of age and over [7,8].

The whipping of animals for public entertainment and financial gain, some of which entails problem gambling is problematic from moral standpoints [1,9,10]. Ethically, there are members of the public who perceive the whipping of racehorses to be cruel [11,12]. Their concerns can be contextualized within broader socio-historical trends in the education of animals *and* humans away from corporal punishment [13,14] and towards positive reinforcement [15]. Indeed, considering the use of an aversive stimulus such as whipping as “encouragement” is more akin to models of corporal punishment now largely abandoned as they are considered outdated, unethical and ineffective [13]. Contemporary methods of human education and animal training are increasingly based on positive reinforcement, which is both socially acceptable and successful [16]. These social, moral and ethical dimensions of whipping could seriously undermine the industry’s SLO [17]. More importantly, animal behaviourists question the assumptions that whip use (1) “encourages” horses to run faster for longer, especially when applied to tired horses, and (2) improves steering, which minimizes interference and therefore increases safety.

Aversive stimuli such as whip use have a role in horse training. However, to be considered ethical they must be applied conservatively and removed with impeccable timing, as articulated in the International Society for Equitation Science’s (ISES) position statement on aversive stimuli in horse training [18], where the removal of the aversive is more important than the application. The idea of an aversive stimulus such as a whip acting as encouragement to go faster is not supported by learning theory [19]. To align horse training with horse ethology, equitation scientists recommend the use of only one response per signal [20]. As such, the horse is unlikely to discriminate between whip use to encourage speed when increasing speed is physiologically possible (negative reinforcement) and whip use to discourage slowing when increasing speed is physiologically impossible (positive punishment). However, whip use is greatest in the final stages of a race when a horse is tired, slowing and less physiologically capable of increasing speed [21]. This is entirely counterproductive as it constitutes positive punishment, which is most effective in *discouraging* behaviour [22,23].

Without the predictability provided by clear timing, consistent whip application, immediate whip removal and the horse’s ability to respond, the horse may respond to single or cumulative whip strikes by entering a nervous system state of hyperarousal demonstrated by flight or fight responses, or hypoarousal demonstrated by freezing, during which it is highly unlikely that a jockey will be able to effect any influence over the horse whatsoever [24,25]. These behaviours can all manifest in unpredictable responses, none of which benefit jockey and horse safety [23]. Moreover, whipping in racing is aversive above and beyond the levels considered acceptable by ISES because it can cause pain and distress [26]. Whip use has been associated with falls [27] and fractures [28], probably because tired horses attract whip use and then, however coincidentally, they make proprioceptive errors.

To mitigate the undesirable and counterproductive outcomes of whip use, the international racing industry has implemented various measures [4]. Based on the assumption that whipping works, these measures have included the use of padded whips and increased regulation over when, how and in what quantity the whip can be used [8]. When whip rules were tightened in the latter part of 2011 by reducing the number of strikes, the BHA reported that whip breaches decreased generally as did interferences [29]. However, problems have been identified with the policing of whip rules [9,30] and there are doubts over the effectiveness of padding on whips to spare horses pain when struck [27].

Given that whip use is considered essential for the safety of horse and jockey in races, the reluctance to ban whip use is understandable. In horseracing, the dangers of a fall from height and at speed are compounded in the group ride situation where there is a further risk of being tripped and/or trampled. The ability of a jockey to steer is essential to reduce the risk of one horse blocking, crossing, impeding or otherwise interfering with the path of another. However, there has been no research dedicated to testing the relationship between whip use, steering, safety and integrity during a race. If whips are necessary for steering, one would expect whipping-free (WF) races to be associated with an increase in the number of stewards’ reports with any issues to report, movement on course, interference on course, and jockey-related incidents.

An ideal way to empirically determine associations between whip use, steering and safety is to compare races where whips are used with races where whips are not used. If whip use reduces the likelihood of serious risks to horses and jockeys by improving steering, then an argument could be made for continued use and modification. If whip use is not associated with improved steering, safety or performance, continuation on those bases would be unwarranted and ill-informed, especially in light of the aforementioned empirical evidence and theoretical research suggesting that it is unethical for equids and antithetical to horseracing integrity.

The main aim of this study was to determine the importance of whip use to racing integrity. Specifically, we sought to test the assumption that whip use improves safety by enhancing steering. Testing these assumptions under experimental conditions comparing whipping and whip-free races would be problematic, not least due to the ethical concerns for potentially harming horses or compromising the safety of jockeys. Fortunately, the British Horseracing Authority (BHA) run a “Hands and Heels” series of races for apprentice jockeys. The conditions for Hands and Heels races are the same as for standard whip races (BHA Annual Programme Book), with the following exceptions: the horses must be suitable for apprentices and whips can be held but must not be used unless required to get a reluctant horse moving at the start of a race or for safety reasons. If the whip is used for any reason, it is subject to an enquiry from the stewards. The Hands and Heels races were created to foster best practice in race-riding without a reliance on the whip. They also provide an unprecedented opportunity to analyse the relationship between steering/safety with and without whip use being permitted. In particular, they allow for a comparative study when a rigorous randomized control trial would lack validity and would be unable to be blinded.

## 2. Materials and Methods

Data were provided from an analysis of the official reports written by racing stewards after every race meeting and publicly available online. The role of racing stewards is to enforce the rules of racing, identify breeches of code and conduct and monitor animal welfare. A report is produced for every race at a meeting, even if simply to note “nothing to report”. Stewards’ reports are critical to racing integrity. They record any and every item of importance relating to a race.

A mixed-method comparative research design was most appropriate for analysing our textual data. Whilst the primary data were qualitative, the research question was ultimately quantitative, as was the need to conduct analyses to infer statistical significance [31]. This kind of approach has been used elsewhere in relation to the analysis of naturalistic or free-text survey data [32]. The study design followed a two-stage qualitative-quantitative design, commencing with qualitative coding followed by inferential statistics including logistic regression.

As the study was confined to the analysis of secondary data, which were publicly available, Human Research Ethics Committee approval was not required. Despite the data being in the public domain (https://www.britishhorseracing.com/racing/stewards-reports/), we have replaced the full names of jockeys with their initials when reproducing extracts from stewards’ reports.

### 2.1. Selection of Cases and Matching Cases

We identified 67 whipping-free (WF) races (spanning January 2017–December 2019) for which we were able to case-match 59 whipping-permitted (WP) races, or 88% of all WF races. Matching of WF races to standard whip races was undertaken to ensure that the control races were similar to the WF races for all variables that may be related to incidents in stewards’ reports. To qualify as an acceptable match, the race had to meet the following criteria: took place between January 2017 and December 2019; took place at the same racecourse; was not a National Hunt (“jump”) race; took place over the same distance; included the same number of horses; and was of a similar race class and going (i.e., track condition) to the WF race. Race class was allowed to deviate by one class, e.g., a class 6 race could be matched with a class 5 or class 7, but not a class 4. Going assessed on turf courses was supplied on a 6 point scale (firm, good to firm, good, good to soft, soft and heavy). Matches had to be no more than 3 units away on this scale, e.g., a good-to-firm race could be matched with a race on soft surface but not on a heavy surface. In most cases, matches had the same going (80%, *n* = 31 of 59), and over a quarter (27%, *n* = 16 of 59) were within one unit of the matched race.

If more than one match was available, the race that took place on the closest date to the WF race was selected as the match. When two WF races were undertaken on the same day, the later race was matched first with the closest date and the earlier race was matched with the next closest date. WF races that could not be matched according to the criteria were excluded from the analysis. This typically involved longer race distances for the course in question, or heavy-going conditions.

The case matching selection criteria generated a total data set of 126 stewards’ reports covering 67 WF and 59 WP races, all of which were flat races run on turf and all-weather (artificial) surfaces in Great Britain. Our total data set of 126 races involving 1178 horse/jockey starters represents the largest sample size possible for this study design.

### 2.2. Qualitative Coding

Stewards’ reports for all 126 races in the data set were downloaded from the internet during April 2020 and imported into the qualitative data analysis program MAXQDA 2020 (VERBI Software GmbH, Invalidenstraße 74, 10557 Berlin, Germany). The length of reports from each race in the data set ranged from one sentence to several paragraphs. The coding process was consistent with the basic tenets of rigorous qualitative data analysis: immersion, coding, categorisation and the generation of themes [33], although theme generation in the current study was largely deductive as it was framed against racing integrity.

Stewards’ reports were subject to four stages of data analysis. In stage 1, each report was coded systematically to identify each unit of information about which stewards reported. Coding was conducted inductively to counter the potential for selection-bias over what parts of the reports were relevant, and to avoid confirmation bias over any unconscious expectation that WF races would be associated with more safety concerns. Consistent with “open coding” [34], codes were phrased naturalistically to preserve the stewards’ naturalistic field observations. Coding to multiple categories was necessary to preserve the complexity of single events detailed in the reports.

Once all 126 reports had been coded, stage 2 involved aggregating the resulting free codes into seven thematic categories (movement on course, horses, jockeys, interference, equipment, horse behaviour and welfare), leaving one free code remaining (“nothing to report”).

In stage 3 of data analysis, the coder (author 1) presented the coding categories systematically to Author 3, who is an expert on racing terminology. Author 3 was asked to agree or disagree with the coder’s decisions about the organising categories. Where there was disagreement, the two researchers returned to the stewards’ reports to view the coded sections in context. This phase resulted in some slight refinement and movement between codes. For example, two codes that had been categorised under “movement on course” were moved to the category of “horses” because they related only to movement out of the stalls (deemed beyond the present scope of incidents on course). In stage 4, the final coding structure of six categories was presented to all authors for discussion and approval. A deductive process was then undertaken to identify the categories relevant to the cultural assumptions that whip use is necessary for steering, safety and countering fatigue in the later stages of a race.

Two categories that were important, but peripheral to the aim of the present article were: “equipment” and “horses”. The category “equipment” contained reports of saddle slippages, lost horseshoes and tongue ties. The category, “horses” contained a variety of horse behaviours to the post and in the stalls, horses deemed unsuitable for apprentices, and some veterinary issues such as nosebleeds (3 races), stopping quickly (3 races), breathing problems (1 race), ringworm (2 races) and lameness (2 races). The four categories selected for presentation in the present article were “nothing to report”, “movement on course”, “interference on course” and “incidents relating to jockey behaviour”. As detailed below, finishing times were also analysed via a wholly quantitative process of data analysis.

### 2.3. Logistic Regression

The statistical significance of findings produced by the qualitative coding process was evaluated with logistic regression testing. Occurrences of one or more coded incident in a race were modelled using logistic regression using the stats package of R [35]. Dependent variables modelled were “movement on course” codes, “interference” codes, “jockey-related incident” codes and the “nothing to report” code. Each of these variables were separately modelled using candidate explanatory variables including matching variables of racecourse, date of race, distance of race (in furlongs, each equivalent to 201.168 m), number of horses in race, class of race and the going of the course at the time of the race. Whether the course runs clockwise or anticlockwise was also considered as a potential explanatory variable.

In the first stage of modelling, any explanatory variable with a *p* value less than 0.2 in a univariate model for that dependent variable was forced into the model, as was whether the race was a case (WF) or a case-matched control (WP). The Akaike information criterion (AIC) of this multivariable model was determined and compared to the AIC of multivariate models with each an explanatory variable with a p value greater than 0.2. If the AIC was reduced by adding an additional explanatory variable, the new explanatory variable was added to the model and the process was repeated.

Once the final model was selected, the linear relationship between the variable and the logit of the outcome and the continuously varying explanatory variables (distance of race, date, and number of horses) were examined gradually and logarithmic, square root and power terms were trialled as appropriate improve fit as measured by AIC.

Finally, odds ratios for WP races compared to WF races were calculated from the coefficients calculated from the selected model. Interpretation of coefficients for variables upon which matching occurred is not appropriate.

The final model for movement on course included the variables case/control, date of Race, class of race and number of horses in the race (log transformation). The AIC of this model was 155.95.

The final model for interference included the variables case/control, date of race, class of race, going of the course at the time of the race, whether the course runs anticlockwise, and number of horses in the race (log transformation). The AIC of this model was 163.01.

The final model for anything to report included the variables case/control, date of race, racecourse nested within whether the course runs anticlockwise, distance of race and number of horses in the race (log transformation). The AIC of this model was 95.014.

The final model for jockey incident included the variables case/control, date of race, going of the course at the time of the race + number of horses in the race (square transformation) + number of horses in the race. The AIC for this model was 86.417.

Models were checked for influential values by checking the standardised residuals and cook distance using the broom package [36] and for multicollinearity by examining variance inflation factors using the car package [37].

### 2.4. Linear Mixed Model Regression

The effect of whip use on finishing time was studied using a linear mixed model regression with the lme4 package [38], setting untransformed race time as the dependent variable, with whip use as a fixed dependent variable, as well as a random variable coding the matched pair to which the race belonged. While inferences cannot be made on the effect of matching variables (race class, number of runners, race distance, course location and course going) on race times, these variables were still fitted, to explain variance between the matched pairs, as matched pairs. Residuals were examined graphically.

## 3. Results

### 3.1. Overall Queries to Race Integrity

Stewards’ reports record any details that might undermine the integrity of a race. Of the 126 stewards’ reports analysed for this study, 18 were marked as having “nothing to report”, of which 12 were from WF races and six were from WP races.

### 3.2. Movement on Course

Instances of horses moving left, moving right or moving both ways were coded 68 times across the full data set of stewards’ reports. Of those instances, 31 were coded in stewards’ reports from WF races and 37 were coded in the stewards’ reports from WP races. One horse was reported to be moving both ways in a WP race (excluded from the data in Table 1 and Table 2).

There were 34 instances of horses moving to the left, of which 15 instances were coded from WF races and 19 were coded from the WP races, as shown in Table 1.

There were 33 instances of horses moving to the right, of which 16 were coded from WF races and 17 from WP races (see Table 2).

### 3.3. Interference on Course

There were 61 incidents of interference identified across all the stewards’ reports, of which 37 (61%) related to WP and 24 (39%) related to WF races. Each form of interference relates to the actions of one horse/jockey affecting another on the course, which may or may not involve physical contact between horses.

As noted above, the coding protocol permitted multiple codes from one unit of text (usually a sentence or paragraph). The interdependence of forms of interference (including an incident of a whip being used in a WF race) is illustrated in the following extract from WF 36 (note that “manoeuvred right” was counted only once in this extract, despite being mentioned twice, as it related to a single incident):

“An enquiry was held to consider interference inside the first furlong when BOMBSHELL BAY, unplaced, ridden by [MC], manoeuvred right-handed causing WICKED SEA (IRE), placed fourth, ridden by [SC], to have to be heavily eased, which in turn interfered with SUMMERSEAT MIST (IRE), placed eighth, ridden by [KS], who in turn interfered with FACE LIKE THUNDER, placed fifth, ridden by [WC], on the rail. [MC] was suspended for five days for careless riding as he had manoeuvred right, causing considerable interference on his inside [MC], the rider of BOMBSHELL BAY, unplaced, had appeared to use his whip contrary to the conditions of the race [MC] and was suspended for seven days for improper riding”.

There were 11 different ways in which interference was described in the current stewards’ reports, as listed in Table 3. Furthermore, there were three forms of interference that undeniably involved physical contact between horses (marked in Table 3 with an asterisk).

### 3.4. Jockey Behaviour

Six codes comprising 21 incidents were created to categorise stewards’ comments on jockeys, which were relevant to the research aims, of which 12 related to WF races and nine to WP races. Thirteen of these incidents related to claims of and charges for careless and/or improper riding, of which eight were reported for WF races and five for WP races. Eight incidents related to jockeys “dropping their hands” (indicative of not pushing the horse to run on, *n* = 1, WP), a jockey fall on course (*n* = 1, WP), and whip use (*n* = 6).

### 3.5. Logistic Regression

Odds ratios for reports in a WP race, compared to the control of a WF race, are shown in Table 4. WP races were not at significantly lower odds for reported incidents, movement on the course, interference or jockey behaviour.

### 3.6. Race Times Linear Mixed Model Regression

**Figure 1:**
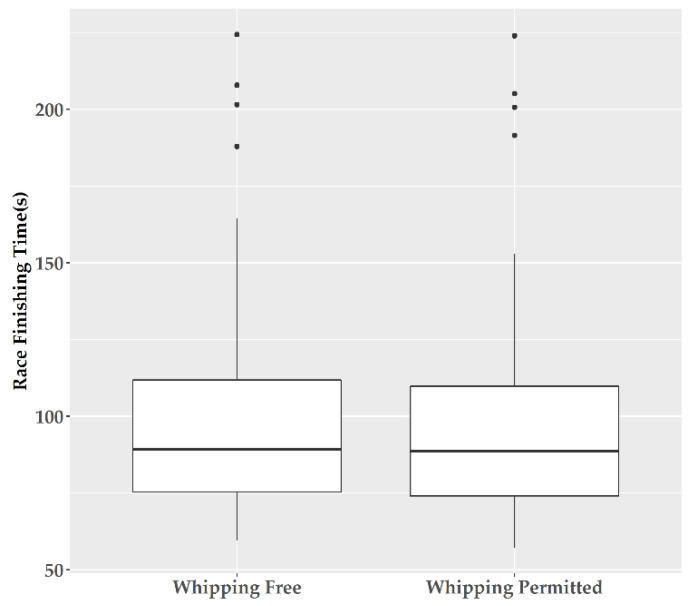
Finishing times for horse races in Great Britain matched for racecourse, distance, class, field and course conditions, with the use of whips permitted and not permitted.

As illustrated in Figure 1, linear mixed model regression on the finishing times of the study races did not show a significant effect of WF racing compared to WP racing (estimate = −0.512 seconds for WP races compared to WF races; standard error = 0.351 seconds; degrees of freedom = 60.647, *t* value = −1.459 *p* = 0.150). The remaining factors in the model (see Materials and Methods, above) were matching variables and therefore their effect on finishing times of horse races more generally cannot be determined.

## 4. Discussion

The aim of this study was to determine if whip use is important to racing integrity by testing the cultural assumption that whip use in racing is necessary for steering and safety. We compared all 67 “Hands and Heels” races run between January 2017 and December 2019 with 59 case-matched whipping races to determine if there was any relationship between safety, steering and whip use.

There were no statistically significant differences in stewards having anything to report or reporting jockey-related behaviour, which would have indicated safety concerns. Neither did we find a significant difference between movement on course or interference in WF and WP races. In other words, we found no evidence that whip use improves steering or reduces interference.

Although not statistically significant, the higher number of reports of horses hanging left in WP races and hanging right in WF races warrants a comment. This difference could be explained by the majority of jockeys holding their whips in their right hand [39] whereby (a) use of the whip in WP races causes horses to veer away from the pressure, which some jockeys have described as dangerous [40] and/or (b) that right-handed jockeys are more likely to steer a horse to veer to the right as a result of biased tension in the right rein, which in WF races is not countered by (a).

The impact of one jockey’s handedness may not even be limited to their own mount, their use of a whip may impact the direction of an adjacent horse. As jockeys sometimes change their whip hand during a race [41], further statistical testing combined with visual analysis of the moment of deviation in movement and application of the whip is required to investigate relationships among whip use, jockey handedness, horse motor laterality [42], steering and direction (especially regarding hanging left/right) and their primary and secondary impacts on the field of horses.

Still, only one study has concluded that whip use can aid steering and that conclusion was not borne out by the data presented [41]. The need to otherwise improve steering therefore seems urgent. We therefore recommend investment in science-based foundation training of racehorses to take advantage of non-whip related cues at jockeys’ disposal, such as the use of an open rein and/or weight shifts. Developments in the pre-training of racehorses could mitigate the natural motor laterality in individual horses [43,44] through tailored interventions to reduce lateral biases in steering. Improving the foundation training of racehorses could have secondary benefits by making ex-racehorses easier to “rehome” after retirement from racing and therefore less represented among wastage figures [45].

Finally, there were no statistically significant differences in the finishing times between WF and WP races. Our findings about race times support other studies that have been unable to associate whip strikes with “significant variation in velocity as a predictor of superior placing at the finish” [21]. It is also consistent with a study on harness racing that found that the reduction in whip use resulted in slightly faster race times and a later reinstatement of whip use showed no significant difference to race times [46]. Our findings undermine the popular assumption that whipping increases the speed of horses, or at least reduces the loss of speed that can be expected towards the end of a race when horses are fatigued.

Interpretation of the results of this study must be undertaken in full consideration of some notable limitations related to sample size, validity and methods. The sample size may appear small relative to the number of races run on the flat in Great Britain each year (over 6000). However, our study covers the past three years and relates to current policy, regulation and penalties. We analysed all 67 WF races (100%) in the study period, of which we were able to case-match 88%. As such, our total data set of 126 races involving 1178 horse/jockey starters represents the largest sample size possible for this study design. The continuation and expansion of whipping-free races such as “Hands and Heels” would provide a larger sample for further analysis, which did not exist during the study period reported in this article.

In relation to validity, the stewards’ reports were taken at face value as true, accurate and exhaustive accounts of the occurrences in each race. After all, they are the most formal records of a race. Whilst stewards’ reports are not immune from human biases and intentional/unintentional errors of omission/commission [47], we suggest that these risks have been mitigated by rigorous steward training and monitoring imposed by the industry. The qualitative coding stage of data analysis was subject to the standard limitations whereby the coder applied some subjective decision-making during the analysis of stewards’ reports. Whilst the chance of a coding error cannot be eliminated, the codes and their categories were verified among the research team, which included a subject expert. The triangulation of visual analysis of race footage with stewards’ reports could be considered in future research to increase validity.

In relation to methods, there were limitations to the sampling strategy as well as the qualitative coding. Our selection of case-matched races does not hold the typical weight ascribed to a control group, although an experimental study design would have been an inappropriate choice for the reasons outlined above. Despite this naturalistic study revealing no relationship between whip use and steering, experimental research on whip use and steering could provide an important source of methodological triangulation.

Our case-matching method was unable to control for all variables. It did not include the location on the course where deviations occurred. Neither did it consider whether on-course movement took place within the last 100 m or over a longer distance, such as within the final furlong. This would be a valuable focus of future racing footage analysis, which could consider the impact of horse fatigue on movement left and/or right as well as other components of racing integrity such as horse/jockey safety and horse welfare, especially regarding the whipping of tired horses [21]. Future research could also identify if direction of travel or particular racecourses exhibit a course bias that may accentuate any tendency for a bias in horse movement or jockey steering left/right.

Overall, our findings—that whip use does not improve steering, reduce interference, increase safety or improve safety—suggest that whip use is not essential to racing integrity. The continuation of WF races would provide further data to address the current study’s limitations and enable the identification of any difference in stewards’ reporting over time (as they become more accustomed to the idea of WF races), or between different racecourses/regions. From a cost-benefit analysis approach to equine welfare [48], any costs to introducing WF races would be exceeded by the benefits to racing integrity, horse welfare, public perception and the industry’s SLO.

## 5. Conclusions

Our comparison of whipping-free and whipping-permitted races found no statistically significant differences in movement on course, interference on course, incidents related to jockey behaviour or race finishing times. That is, there was no evidence that the use of whips contributed to steering, reduced the likelihood of interference, improved the safety of horse or jockey or made horses run faster overall. Our findings refute the culturally entrenched belief that whip use is essential for racing integrity, particularly in relation to steering, safety and riding the horse out on its merits. In other words, we found nothing to commend the use of the whip in horseracing that could (a) be related to integrity, (b) counter the scientific evidence that whip use is a welfare concern or (c) alleviate increasing public discontent with horseracing. As such, whipping-free races could be adopted more broadly by the industry internationally without compromising racing integrity or horse/jockey safety.

## Figures and Tables

**Table 1:** The 34 instances of horses moving to the left, as described in the stewards’ reports (excluding the one instance of a horse moving both ways).

Type of Movement Left	WF Races	WP Races	Total
Hung left	6 (38%)	10 (62%)	16 (47%)
Drifted left	3 (50%)	3 (50%)	6 (18%)
Shifted left	2 (50%)	2 (50%)	4 (12%)
Edged left	2 (50%)	2 (50%)	4 (12%)
Manoeuvered left-handed	2 (100%)	0 (0%)	2 (6%)
Lugged left	0 (0%)	1 (100%)	1 (3%)
Steered left-handed	0 (0%)	1 (100%)	1 (3%)
Movement left	15 (44%)	19 (56%)	34 (100%)

**Table 2:** The 33 instances of horses moving to the right, as described in the stewards’ reports, (excluding the one instance of a horse moving both ways).

Type of Movement Right	WF Races	WP Races	Total
Hung right	12 (63%)	7 (37%)	19 (58%)
Edged right	2 (33%)	4 (67%)	6 (18%)
Shifted right	0 (0%)	3 (100%)	3 (9%)
Drifted right	1 (50%)	1 (50%)	2 (6%)
Lugged right	0 (0%)	2 (100%)	2 (6%)
Manoeuvered right-handed	1 (100%)	0 (0%)	1 (3%)
Movement right	16 (50%)	17 (50%)	33 (100%)

**Table 3:** The 61 instances of interference as described in the stewards’ reports. The asterisk (*) denotes undeniable physical contact between horses.

Form of Interference	WF Race	WP Race	Total
Interference (verbatim/undefined or prior to elaboration)	9 (38%)	16 (67%)	25 (41%)
Caused another horse to be checked/eased	5 (38%)	8 (62%)	13 (21%)
Caused another horse to lose line	2 (25%)	6 (75%)	8 (13%)
Caused another horse to be hampered	3 (75%)	1 (25%)	4 (7%)
* Clipped heels	2 (67%)	1 (33%)	3 (5%)
Caused another horse to be tightened for room	2 (67%)	1 (33%)	3 (5%)
Caused another horse to veer sharply	0 (0%)	1 (100%)	1 (2%)
* Horse struck into	0 (0%)	1 (100%)	1 (2%)
Denied a clear run	0 (0%)	1 (100%)	1 (2%)
* Bumped another horse	0 (0%)	1 (100%)	1 (2%)
Crowded another horse	1 (100%)	0 (0%)	1 (2%)
Totals	24 (39%)	37 (61%)	61 (100%)

**Table 4:** The odds ratios, z values, p values and 95% confidence intervals for reports in a WP race, compared to the control of a WF race where whips are held but not used.

Category of Analysis	Odds Ratio	*Z* Value	*p* Value	Lower 95% Confidence Interval Limit	Upper 95% Confidence Interval Limit
Anything to report	3.06	1.500	0.134	0.74	14.73
Any movement on course	1.10	0.221	0.825	0.48	2.49
Interference	0.90	−0.223	0.824	0.37	2.17
Jockey behaviour	1.24	0.689	0.316	0.32	5.07
